# 4,4′-methylenediphenol reduces Aβ-induced toxicity in a *Caenorhabditis elegans* model of Alzheimer’s disease

**DOI:** 10.3389/fnagi.2024.1393721

**Published:** 2024-05-30

**Authors:** Xingzhi Yu, Jie Tao, Tian Xiao, Xiaohua Duan

**Affiliations:** Yunnan Key Laboratory of Dai and Yi Medicines, Yunnan University of Chinese Medicine, Kunming, Yunnan, China

**Keywords:** 4,4′-methylenediphenol, Alzheimer’s disease, *Caenorhabditis elegans*, antioxidant activity, Aβ protein, metabolomics

## Abstract

**Introduction:**

*Gastrodia elata Blume* is a widely used medicinal and edible herb with a rich chemical composition. Moreover, prescriptions containing *Gastrodia elata* are commonly used for the prevention and treatment of cardiovascular, cerebrovascular, and aging-related diseases. Recent pharmacological studies have confirmed the antioxidant and neuroprotective effects of *Gastrodia elata*, and, in recent years, this herb has also been used in the treatment of Alzheimer’s disease (AD) and other neurodegenerative disorders. We have previously shown that 4,4′-methylenediphenol, a key active ingredient of *Gastrodia elata*, can mitigate amyloid-β (Aβ)-induced paralysis in AD model worms as well as prolong the lifespan of the animals, thus displaying potential as a treatment of AD.

**Methods:**

We investigated the effects of 4,4′-methylenediphenol on AD and aging through paralysis, lifespan, and behavioral assays. In addition, we determined the anti-AD effects of 4,4′-methylenediphenol by reactive oxygen species (ROS) assay, lipofuscin analysis, thioflavin S staining, metabolomics analysis, GFP reporter gene worm assay, and RNA interference assay and conducted in-depth studies on its mechanism of action.

**Results:**

4,4′-Methylenediphenol not only delayed paralysis onset and senescence in the AD model worms but also enhanced their motility and stress tolerance. Meanwhile, 4,4′-methylenediphenol treatment also reduced the contents of reactive oxygen species (ROS) and lipofuscin, and decreased Aβ protein deposition in the worms. Broad-spectrum targeted metabolomic analysis showed that 4,4′-methylenediphenol administration had a positive effect on the metabolite profile of the worms. In addition, 4,4′-methylenediphenol promoted the nuclear translocation of DAF-16 and upregulated the expression of SKN-1, SOD-3, and GST-4 in the respective GFP reporter lines, accompanied by an enhancement of antioxidant activity and a reduction in Aβ toxicity; importantly, our results suggested that these effects of 4,4′-methylenediphenol were mediated, at least partly, via the activation of DAF-16.

**Conclusion:**

We have demonstrated that 4,4′-methylenediphenol can reduce Aβ-induced toxicity in AD model worms, suggesting that it has potential for development as an anti-AD drug. Our findings provide ideas and references for further research into the anti-AD effects of *Gastrodia elata* and its active ingredients.

## Graphical abstract

In this study, we investigated the anti-AD efficacy and mechanism of 4,4′-methylenediphenol, an active ingredient of *Gastrodia elata*, using the *C. elegans* model. Our study showed that 4,4′-methylenediphenol enhances the expression of DAF-16, SOD-3, SKN-1, and GST-4 by activating the DAF-16/FOXO and SKN-1/NRF2 signaling pathways and positively affects metabolites. These combined effects enhance antioxidant stress capacity, which in turn reduces ROS and Aβ aggregation, decreases Aβ toxicity and thus delays neuroparalysis, improves mobility and stress resistance, and ultimately achieves anti-AD effects. (The figure was drawn By Figdraw: www.figdraw.com).

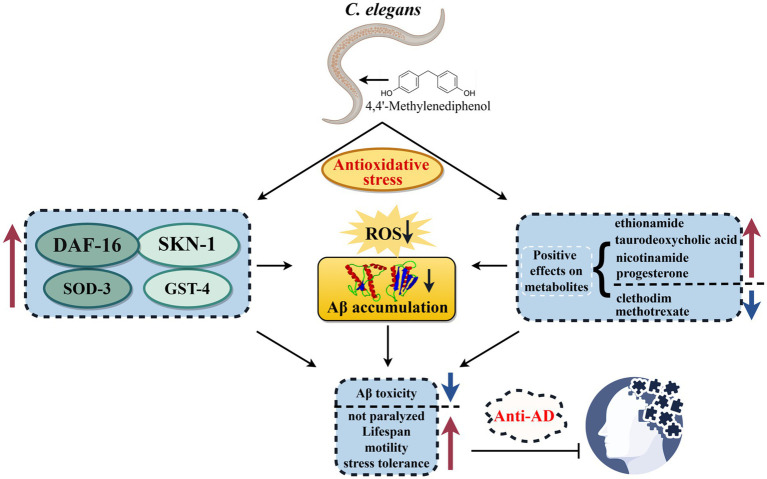

## 1 Introduction

Alzheimer’s disease (AD) is an irreversible age-related neurodegenerative disorder with an insidious onset and a long-term course. Affecting mostly older adults, the main clinical manifestations include cognitive deficits, mental and behavioral abnormalities, and impaired sociability. AD accounts for the highest proportion of dementia-related diseases (Knopman et al., 2021; Stocker et al., 2023). With the aging of the world’s population, AD and other age-related disorders are increasingly becoming a global health concern (Jensen et al., 2020). Over 50 million people are thought to be living with AD worldwide, and this number is expected to triple to 152 million by 2050 (Weuve et al., 2014). The pathogenesis of AD is complex. The β-amyloid degradation toxicity hypothesis is currently the main theory explaining the pathophysiology of this condition. This hypothesis suggests that abnormal amyloid-β (Aβ) aggregation in the brain is the main triggering factor for AD and that this aggregation can exert direct toxic effects on neuronal cells, triggering a cascade of tau protein phosphorylation, inflammation, excitotoxicity, and oxidative stress, ultimately inducing neuronal death (Alafuzoff et al., 2008; Mohsenzadegan and Mirshafiey, 2012). Accordingly, it is expected that inhibiting Aβ aggregation and improving the body’s antioxidative capacity represent promising therapeutic strategies for the prevention and management of AD. However, there is currently no treatment for this debilitating condition, and the available drugs are mainly used to improve disease symptoms. Accordingly, there is an urgent need for identifying and developing novel drugs aimed at the prevention and treatment of AD (Tan W. et al., 2022).

The effective natural compounds used in traditional Chinese medicine (TCM) and ethnomedicine have advantages such as abundant resource availability, high development potential, and wide-ranging pharmacological activities. *Gastrodia elata* Blume is a perennial herb within the family Orchidaceous and is one of the most valuable TCMs (Huang, 1985). In the “Compendium of Materia Medica,” *Gastrodia elata* is listed as having the effects of calming the liver and suppressing liver Yang, dispelling wind and clearing channels, and stopping convulsions, and it is mainly used for the treatment of paralysis, trance, and aphasia in the state of shock (Tao et al., 2023). Indeed, *Gastrodia elata* has long been used in Chinese folk medicine for the prevention and treatment of neurological diseases that display symptoms similar to those of AD, such as amnesia, intellectual disability, hemiplegia, and speech disorders. Moreover, a group of well-known prescriptions containing *Gastrodia elata*, such as *Gastrodia (Tianma)* and *Uncaria* (*Gouteng*) decoction, have documented antioxidant, free radical scavenging, and neuroprotective effects, and have been used for the prevention and treatment of cardiovascular, cerebrovascular, and aging-related diseases (Liu and Mori, 1992; Deng et al., 2020). Recent pharmacological studies have also found that *Gastrodia elata* has the effects of promoting brain health, protecting nerves, and delaying aging. Additionally, *Gastrodia elata* has greater efficacy and is more widely used than other TCMs with similar effects (e.g., *Ginseng*, *Astragalus membranaceus*), and has recently been applied for the treatment of AD and other related diseases in the clinic (Lu et al., 2023). Meanwhile, studies have shown that the ethyl acetate extract of *Gastrodia elata* (EEGE) has both neuroprotective and cerebral protective effect, and can improve the symptoms of AD in a nematode model of the condition by mitigating Aβ toxicity and oxidative stress (Shi et al., 2023b). In our preliminary study, we isolated a major component of EEGE, 4,4′-methylenediphenol, which is a bisphenol derivative with antioxidant activity (Yi-Ming et al., 1993; Tang et al., 2016). We found that 4,4′-methylenediphenol not only delayed Aβ protein-induced paralysis in AD model worms but also prolonged their lifespan, suggesting that it has the potential for use in the prevention and treatment of AD.

*Caenorhabditis elegans* is a widely used model animal for the study of age-related neurodegenerative diseases. It has the advantages of a short life cycle, small size (~1 mm), ease of cultivation and observation, low costs, freezing and resuscitation ability, and greater availability of transgenic lines compared with other AD model animals (Ewald and Li, 2012; Rani et al., 2023). In addition, *C. elegans* and human genes are highly homologous, and their key cellular metabolic and signaling pathways are highly conserved, making *C. elegans* amenable to *in vivo* studies of AD and other neurodegenerative diseases (Tissenbaum, 2015). Studies have demonstrated the suitability of employing the *C. elegans* model for the screening and development of natural drugs targeting AD. For example, one study found that the extract of *Radix Stellariae* can effectively reduce Aβ and tau protein expression and attenuate the damage they cause, which has therapeutic relevance for neurodegenerative disorders such as AD (Long et al., 2023). In addition, grape juice (Canedo-Reis et al., 2023), carnosic acid (Chen et al., 2022), and wolfberry (Meng et al., 2022) have all been shown to exert anti-AD effects in studies that have used *C. elegans* as a model. In conclusion, *C. elegans* has become an essential model for the screening of AD-targeting drugs.

In the present study, we leveraged the unique advantages of *C. elegans* to investigate the therapeutic efficacy of 4,4′-methylenediphenol against AD as well as the putative underlying mechanisms. First, the potential of this active ingredient as a treatment of AD was investigated in terms of its ability to mitigate Aβ-induced toxicity and oxidative stress, ameliorate AD-associated behavior, and delay aging and senescence. Meanwhile, the ability of 4,4′-methylenediphenol to improve AD symptoms was determined through the determination of ROS and lipofuscin contents and the levels of Aβ deposition. Finally, the mechanism underlying how 4,4′-methylenediphenol exerts its anti-AD effects was investigated in depth using broad-spectrum targeted metabolomics (TM) analysis, GFP reporter lines, and RNAi. Our findings provide a reference for further basic research into the therapeutic potential of the active ingredients of *Gastrodia elata* in AD prevention and treatment.

## 2 Materials and methods

### 2.1 Materials

4,4′-methylenediphenol was purchased from Chengdu Alfa Biotechnology Co. Ltd. (purity: 98%; 620–92-8). The worms were grown in nematode growth medium (NGM) containing sodium chloride, peptone, agar powder, cholesterol, anhydrous calcium chloride, anhydrous magnesium sulfate, and phosphate buffer. RNAi plates were prepared by adding ampicillin (96%; 7,177-48-2) and isopropyl-beta-D-thiogalactopyranoside (GC >8%; 367–93-1; Shanghai Macklin Biochemical Co., Ltd., China) to final concentrations of 100 μg/mL and 1 mM, respectively. To inhibit worm egg-laying, 5-fluoro-2′-deoxyuracil nucleoside (floxuridine, FUDR; 50–91–9; Shanghai Macklin Biochemical Co., Ltd., China) was added to the medium to a final concentration of 12 μM. The anesthetic levamisole hydrochloride (HPLC ≥98%; 16,595–80-5) was purchased from Sichuan Weikeqi-biotech Co. Ltd.; SuperKine Enhanced Antifluorescence Quencher (BMU104-CN) was supplied by Abbkine Scientific Co., Ltd.

### 2.2 *Caenorhabditis elegans* strains and maintenance

The *C. elegans* lines used in this study were CL4176 {dvIs27 [myo-3p::A-Beta (1–42)::let-851 3′UTR) + rol-6(su1006)] X.}, CL2006 {dvIs2 [pCL12(unc-54/human Abeta peptide 1–42 minigene) + rol-6(su1006)]}, TJ356 {zIs356 [daf-16p::daf-16a/b::GFP + rol-6(su1006)]}, LD1 (ldIs7 [skn-1b/c::GFP + rol-6(su1006)]}, CF1553 {mu1s84[(pAD76) sod-3p::GFP) + rol-6(su1006)]}, CL2166 {dvIs19 [(pAF15) gst-4p::GFP::NLS] III}, and *C. elegans* N2 (wild-type strain). All worm strains were purchased from the *C. elegans* Genetics Center (CGC, University of Minnesota, Minneapolis, MN, United States). An uracil synthesis-deficient strain of *Escherichia coli* (OP50) was used as food. *C. elegans* CL4176 was grown and reproduced at 16°C; all other strains were grown and reproduced at 20°C.

### 2.3 Paralysis assay

4,4′-Methylenediphenol was weighed on an electronic precision balance, dissolved in deionized water [with DMSO (0.2%) as co-solvent] to a final concentration of 10 mM, filtered to remove bacteria, and stored at 4°C until needed. Different concentrations of the drug (0.125, 0.25, 0.5, 1, 2, and 4 mM) were generated by diluting the drug master mix with a solution containing inactivated OP50 bacteria (OD600 = 0.6, 30 min at 65°C). After mixing well, the mixture was added to the surface of the NGM, and allowed to air-dry before use. Worms in the Control group were given an equal volume of the bacterial solution. Subsequently, L1 stage-synchronized CL4176 worms were transferred to NGM plates with or without the drug (30–50 worms per plate). After incubation to the L3 stage at 16°C, the temperature was raised to 25°C, and incubation was continued for approximately another 24 h. The number of paralyzed worms was recorded every 2 h until all the worms were paralyzed. Paralysis was defined as rigid movement or only head movement following mechanical stimulation. The optimal effective concentration of the drug as determined by the paralysis assay was used the drug concentration in subsequent experiments.

### 2.4 Lifespan assay

L4 stage-synchronized CL4176 worms were transferred to plates with or without the drug (1 mM), which was recorded as day 0. FUDR (12 μM) was added to each plate to prevent egg-laying and larval hatching affecting the experimental counts. Three plates were run in parallel for each group (30–50 worms per plate). Thereafter, the number of surviving worms was recorded every 2 days, following which the surviving worms were transferred to fresh medium containing the same drug concentration, and culture was continued until all of the worms had died. Worms that did not respond to stimulation with a picker were considered dead. Worms that developed a bag-like phenotype and did not die within the area coated with the drug were excluded from the statistical analysis. The lifespan assay was repeated three times with no less than 60 worms each time.

### 2.5 Lipofuscin assay

L1 stage-synchronized N2 worms were cultured to the L4 stage at 20°C and then transferred to NGM plates with or without the drug (1 mM). After 10 days of incubation, worms from each group (≥10 worms per group) were transferred to PBS containing anesthetic and analyzed for lipofuscin autofluorescence under a fluorescence microscope (LSM900, Carl Zeiss, Germany). Fluorescence intensity values were determined using ImageJ software. The experiment was repeated independently three times.

### 2.6 Reproductive and behavioral assays

#### 2.6.1 Reproductive capacity assay

CL4176 worms that had not yet performed egg laying when synchronized at the L4 stage were transferred to NGM culture plates with or without the drug (1 mM) (two worms per plate, three plates in parallel per group) and incubated at 16°C; this was recorded as day 1 of reproduction. Every day thereafter, the worms were transferred to new culture plates containing the same drug concentrations, and the number of worms hatched in the old dishes on day 1 was counted on day 3 until the worms no longer produced new eggs. The effect of the drug on the reproductive ability of the worms was determined by recording and totaling the number of larvae hatched per plate in each group.

#### 2.6.2 Behavioral assays, including pharyngeal pump beating and motility

L3 stage-synchronized CL4176 worms were transferred to NGM culture plates with or without the drug (1 mM) (three plates per group in parallel) and incubated at 16°C; this was recorded as day 0. Every second day, the worms were transferred to fresh culture plates containing the same drug concentrations. Pharyngeal pump beating and motility were observed under a stereoscopic microscope on days 4, 6, and 8 after drug treatment. Before observing the swallowing rate, the worms were transferred to new plates and observed again, and the number of times the worms swallowed in 30 s was used as the index of pharyngeal pump beating. The pharyngeal pump beating upward and downward once was considered a complete pharyngeal movement; 10 worms were randomly evaluated in each plate. Before observing motility, the worms were transferred to a new plate with 100 μL of M9 solution, and the number of sinusoidal movements in 30 s was recorded as an indicator of motility; 10 worms were randomly assessed in each plate. The behavioral experiments were repeated three times, and 30 worms were counted in each group each time.

### 2.7 Body length assays

N2 worms synchronized to the L4 stage were transferred to NGM plates (12 μM FUDR) with or without drug (1 mM), and the size of the worms was determined after continued incubation at 20°C for 2 days. Worms were collected with PBS buffer and anesthetized, and at least 20 worms in each group were imaged under an inverted biomicroscope (DMI1, Leica Microsystems (Shanghai) Co., Ltd.), and the pictures were saved. Worm body length (μm) was measured using Image J software. The experiments were repeated three times independently.

### 2.8 Stress tolerance assays

#### 2.8.1 Heat stress assay

N2 worms synchronized at the L1 stage were transferred to NGM culture plates with or without the drug (1 mM) (three plates in parallel, ≥90 worms per group) and incubated at 20°C for 2 days, following which the incubation temperature was increased to 37°C. Thereafter, survival was observed every hour until all the worms had died.

#### 2.8.2 Oxidative stress assay

L1 stage-synchronized N2 worms were transferred to NGM plates with or without the drug (1 mM) and, after incubation to the L4 stage at 20°C, the worms were transferred to NGM plates containing juglone (300 μM; 481–39-0; Shanghai Yuanye Bio-Technology Co., Ltd., China) (three plates per group, ~30 worms per plate). Worm survival was then observed every hour until all the worms had died. Worms that were stiff and motionless and did not respond to light sources or slight vibrations were considered dead. All the experiments were repeated independently three times.

### 2.9 Reactive oxygen species assay

N2 worms synchronized at the L1 stage were transferred to NGM culture plates with or without the drug (1 mM) (~50 worms per plate) and incubated at 20°C to the L4 stage. Then, 5 mM paraquat solution (methyl viologen dichloride hydrate; 1910-42-5; Shanghai Aladdin Biochemical Technology Co., Ltd) was added to the NGM plates for 4 h, after which the worms were collected and washed three times with PBS. Subsequently, 50 μL of 10 μM DCFH-DA solution (Reactive Oxygen Species Detection Kit; S0033S; Beyotime Biotech. Inc.) was added, and the worms were incubated at 37°C for 30 min. After washing three times with PBS, 30 μL of worm-containing fluid was placed on a slide, coverslipped, and observed under a fluorescence microscope (LSM900, Carl Zeiss), and adjust the parameters, save the picture, and measure the fluorescence intensity value using Image J software. At least 10 worms in each group.

### 2.10 Sedimentation assay for Aβ aggregation

L1 stage-synchronized CL2006 worms were transferred to NGM plates with OP50 bacterial solution only, incubated to the L4 stage at 20°C, and then transferred to NGM plates with or without the drug (1 mM). After 2 days of incubation, the worms were collected in M9 buffer, washed three times, and fixed in 1 mL of 4% paraformaldehyde (pH 7.4; BL539A; Labgic Technology Co., Ltd) at 4°C for 24 h. After discarding the fixative, the worms were incubated in 1 mL of permeabilizing solution [5% β-hydrophobic ethanol [60–24-2; Shanghai Macklin Biochemical Co., Ltd], 1% Triton X-100 [9002-93-1; Meilunbio Biotechnology Co., Ltd], and 125 mM Tris [pH 7.4; 77–86-1; Beijing Solarbio Science & Technology Co., Ltd]] at 37°C for 24 h and rinsed three times with TBST solution. Then, 100 μL of the fluorescent dye thioflavin S (0.125%) (1326-12-1; Shanghai Yuanye Bio-Technology Co., Ltd) was added, followed by incubation at room temperature for 2 min. After discarding the staining solution, the worms were cleared twice with 50% ethanol, and Aβ protein deposition in the head of worms (i.e., the part before the pharyngeal pump) was observed under a fluorescence microscope (LSM900, Carl Zeiss). For imaging, 30 μL of worm-containing fluid was aspirated and placed on a slide, coverslipped, and the parameters adjusted. At least 10 worms were assessed in each group.

### 2.11 Broad-spectrum TM analysis

#### 2.11.1 Sample preparation

CL4176 worms synchronized to the L1 stage were transferred to NGM plates with or without the drug (1 mM) (≥1,000 per plate, 6 plates in parallel per group), cultured first at 16°C for 48 h, and then at 25°C for approximately 30 h. The worms were collected in EP tubes with M9 buffer, washed 2–3 times with sterile water, quick-frozen in liquid nitrogen, and stored at −80°C for sequencing.

#### 2.11.2 Broad-spectrum TM analysis

Samples were thawed on ice, added to stainless steel balls, and homogenized with a ball mill (30 Hz) for 20 s. The samples were then extracted with a 70% methanol aqueous solution at 4°C and used for analysis. All operations were performed on ice. Sample analysis, including differential metabolite screening and metabolic pathway resolution, was performed at Wuhan Metware Biotechnology Co., Ltd. First, pooled samples were subjected to non-targeted metabolomics analysis to identify the metabolites in the samples. Subsequently, these metabolites were combined with those of Metware’s in-house metabolite database (MWDB) for broad-spectrum TM analysis. Metabolomic differences between samples were assessed based on a combination of ultra-high performance liquid chromatography–tandem mass spectrometry (UPLC–MS/MS), the in-house database, and multivariate statistical analysis. Chromatographic separation was performed using a Waters ACQUITY UPLC HSS T3 C18 column (1.8 μm, 2.1 × 100 mm); mobile phase A was 0.1% formic acid in ultrapure water and mobile phase B was 0.1% formic acid in acetonitrile; the flow rate was 0.4 mL/min; the column temperature was 40°C; the injection volumes were 2 μL for extensive targeted detection and 5 μL for non-targeted detection. For hydrophilic interaction liquid chromatographic (HILIC) separation, a waters ACQUITY UPLC BEH HILIC column (1.7 μm, 1 × 100 mm) was used; mobile phase A consisted of 20 mM ammonium formate, 30% water, 10% methanol, and 60% acetonitrile, adjusted to pH 10.6 with ammonia; mobile phase B comprised 20 mM ammonium formate, 60% water, and 40% acetonitrile, adjusted to pH 10.6 with ammonia; the column temperature was 40°C; the flow rate was 0.4 mL/min; the injection volumes were 2 μL for broad-spectrum targeted detection and 5 μL for non-targeted detection. For non-targeted metabolomics profiling, data were acquired on a UPLC system^1^ connected to a TripleTOF 6,600 quadrupole time-of-flight (QTOF) mass analyzer (AB SCIEX). For targeted metabolomics profiling, data were acquired on a UPLC system (see text footnote 1, respectively) and a Q-Trap mass spectrometer.^2^ QC samples were formed by mixing equal amounts of all the samples. The QC samples were then analyzed on the LC–QTOF–MS/MS platform and accurately characterized based on MWDB (with secondary spectra and retention time), the AI-predicted library of the MWDB and all public databases (Metlin, HMDB, KEGG, and others), and MetDNA. Multi-ion pair and retention time information for the identified metabolites was also extracted, and this information was combined with the information in Metware’s target database to form a new library exclusive to the project. Finally, for all the samples, the metabolites in the new library were quantified on the Q-Trap instrument in multiple-reaction monitoring mode. The differential metabolite screening criteria were VIP >1 and *p*-value <0.05.

### 2.12 Transgenic *daf-16*, *skn-1*, *sod-3*, and *gst-4* GFP reporter strains

For all gene expression experiments involving *C. elegans* GFP reporter strains, L1-stage worms were incubated on NGM plates with or without the drug (1 mM) for 72 h, transferred to slides, anesthetized, and fixed in 2% levamisole. Images were captured under a fluorescence microscope (LSM900, Carl Zeiss) at 10/40× magnification and analyzed using ImageJ software. As the positive controls for the TJ356 strain, worms were cultured on plates containing blank medium for 72 h, and the temperature was then increased to 37°C for 30 min for induction. SKN-1::GFP fluorescence intensity was measured in the intestinal region of LD1 worms under the pharynx. The nuclear localization of DAF-16 was expressed as the number of fluorescence puncta per TJ356 worm (Kang et al., 2022). Finally, the whole-body fluorescence intensity associated with SOD-3::GFP and GST-4::GFP expression was evaluated in CF1553 and CL2166 worms, respectively. The experiments were independently replicated three times, with a minimum of 10 worms per group per strain.

### 2.13 *Caenorhabditis elegans* RNA interference assay

After synchronization, the resulting eggs of CL4176 worms were transferred to culture plates coated with R13H8.1 *E. coli* containing *daf-16* dsRNA to knock down *daf-16* and incubated at 16°C. Eggs incubated on culture plates coated with the HT115 *E. coli* strain expressing the L4440 empty vector served as controls. After passing to the third generation, the worms were synchronized, and the resulting eggs were placed in M9 buffer and incubated at 16°C. RNAi efficiency was subsequently assessed by qPCR. Worms successfully subjected to RNAi were then transferred to NGM plates coated with a solution of either RNAi-expressing bacteria or the empty vector-expressing strain and with or without the drug (1 mM), and were cultured to the L3 stage at 16°C. Subsequently, the plates were transferred to 25°C for heat shock, which led to the expression of high levels of Aβ protein in the muscle cells of the body wall of the worms. This resulted in continuous Aβ peptide aggregation, leading to muscle paralysis in the worms. After 24 h, the number of paralyzed worms in each group was counted every 2 h until the proportion of paralyzed worms in the blank control group accounted for more than 80% of the total number of worms. Three plates were run in parallel, with a least 90 worms per group. The experiment was independently repeated three times.

### 2.14 Statistical analysis

The data were statistically analyzed and plotted using GraphPad Prism 8.0 and the results are expressed as means ± standard deviation. Differences between and among groups were analyzed by the *t*-test or one-way ANOVA, respectively. Survival was analyzed using the log-rank test. *p*-values <0.05 were considered significant (*p < 0.05, **p < 0.01, ***p < 0.001). The experiments were independently repeated 3 times.

## 3 Results

### 3.1 4,4′-methylenediphenol delayed beta amyloid-induced paralysis

**Figure 1:**
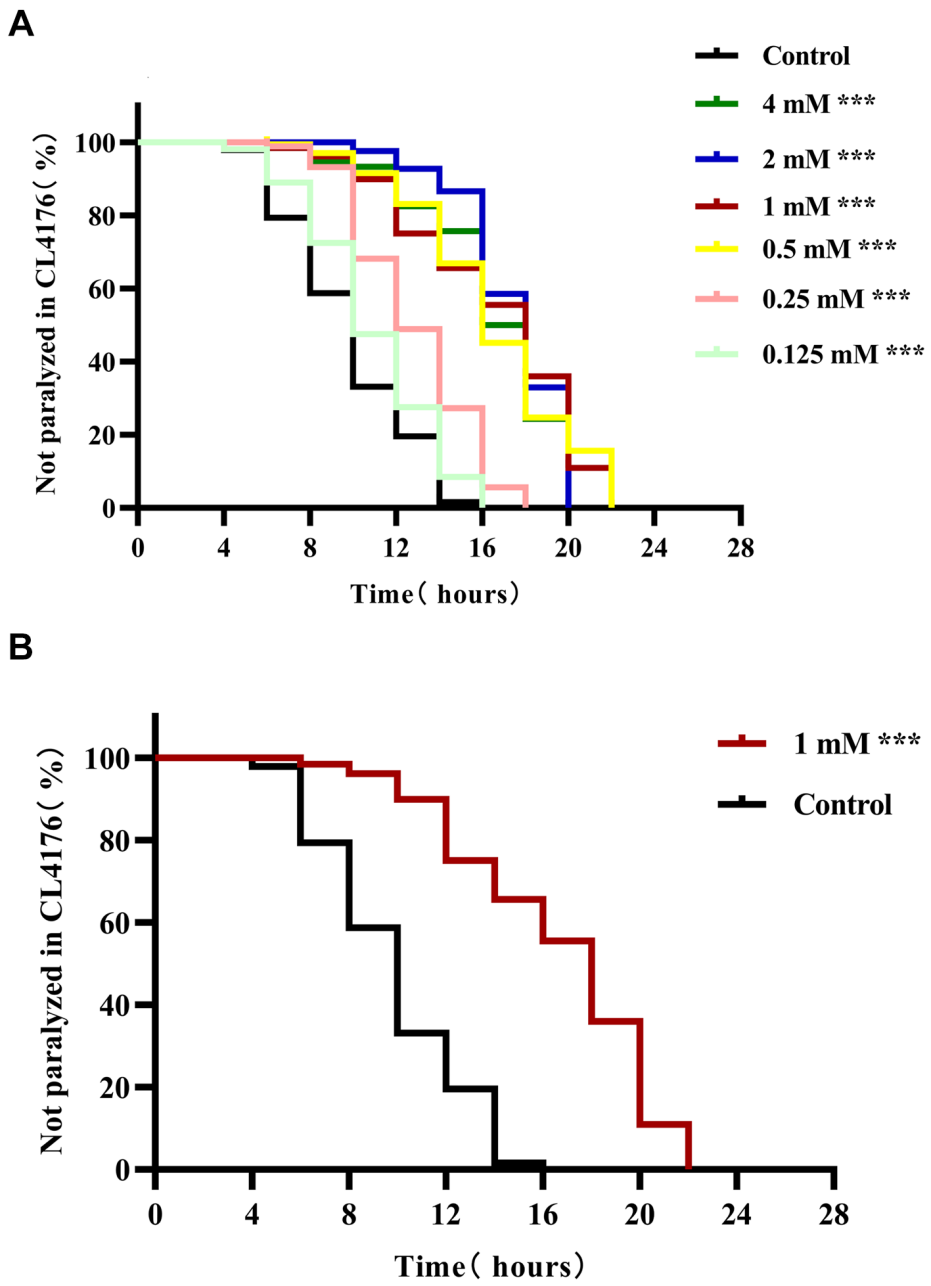
4,4′-methylenediphenol delays paralysis in AD worms. After synchronization to the L1 stage, AD transgenic worms CL4176 were treated with different concentrations of 4,4′-methylenediphenol, respectively, and incubated at 16°C for 48 h with equal volume of OP50 bacterial solution without the drug as a blank control (Control group) and then transferred to 25°C to induce the paralyzed phenotype. **(A)** 4,4′-methylenediphenol treatment delayed β-amyloid-induced paralysis, and although half of the worms were paralyzed for a longer period of time at concentrations of 2 and 4 mM, the drug concentration of 1 mM prolonged the overall paralysis of worms for a longer period of time, and so its optimal concentration was 1 mM (*p* < 0.001). **(B)** Paralysis curves of CL4176 worms under administered or non-administered (1 mM) treatments, with 1 mM administration having the most significant effect in delaying the paralysis of worms (*p* < 0.001).

Under normal conditions, the body of CL4176 transgenic worms is curved (i.e., C-shaped), and the worms can move normally in culture medium. However, when the culture temperature is increased, CL4176 worms express large quantities of the Aβ_1–42_ peptide, which exerts toxic effects on the muscle cells of the animals through aggregation (Sola et al., 2015; Fu et al., 2023). To investigate the potential effect of 4,4′-methylenediphenol on the progression of Aβ toxicity-induced paralysis, we administered different concentrations (0.125, 0.25, 0.5, 1, 2, and 4 mM) of the drug to CL4176 worms and undertook a paralysis assay. The data showed that the time to paralysis was longer in all the 4,4′-methylenediphenol treatment groups than in the Control group (Figure 1A). Except for the group administered the lowest dose (0.125 mM), the time to paralysis for 50% (PT50) of the nematodes was greater in all dosing groups than in the Control group (Table 1). Although the PT50s of the 2 and 4 mM administration groups were longer than that of the 1 mM group, the difference was minimal, and the 1 mM concentration exerted a more significant effect in prolonging the overall paralysis rate of worms (Figure 1B). Based on the principle of selecting the lowest concentration among those with comparable efficacy, 1 mM was deemed to be the optimal effective concentration for this drug and was used for subsequent experiments. In conclusion, the above results indicated that 4,4′-methylenediphenol delayed the onset of paralysis in worms and had a protective effect against β-amyloid-induced toxicity.

**Table 1:** Time to half paralysis for each concentration of drug (*n* = 3).

Drug concentration(mM)	Half of the paralyzed time(PT50; h)
0.125	8.980
0	10.327
0.25	11.152
0.5	14.323
1	14.477
4	14.783
2	15.846

### 3.2 4,4′-methylenediphenol prolonged the lifespan and enhanced the motility of worms

**Figure 2:**
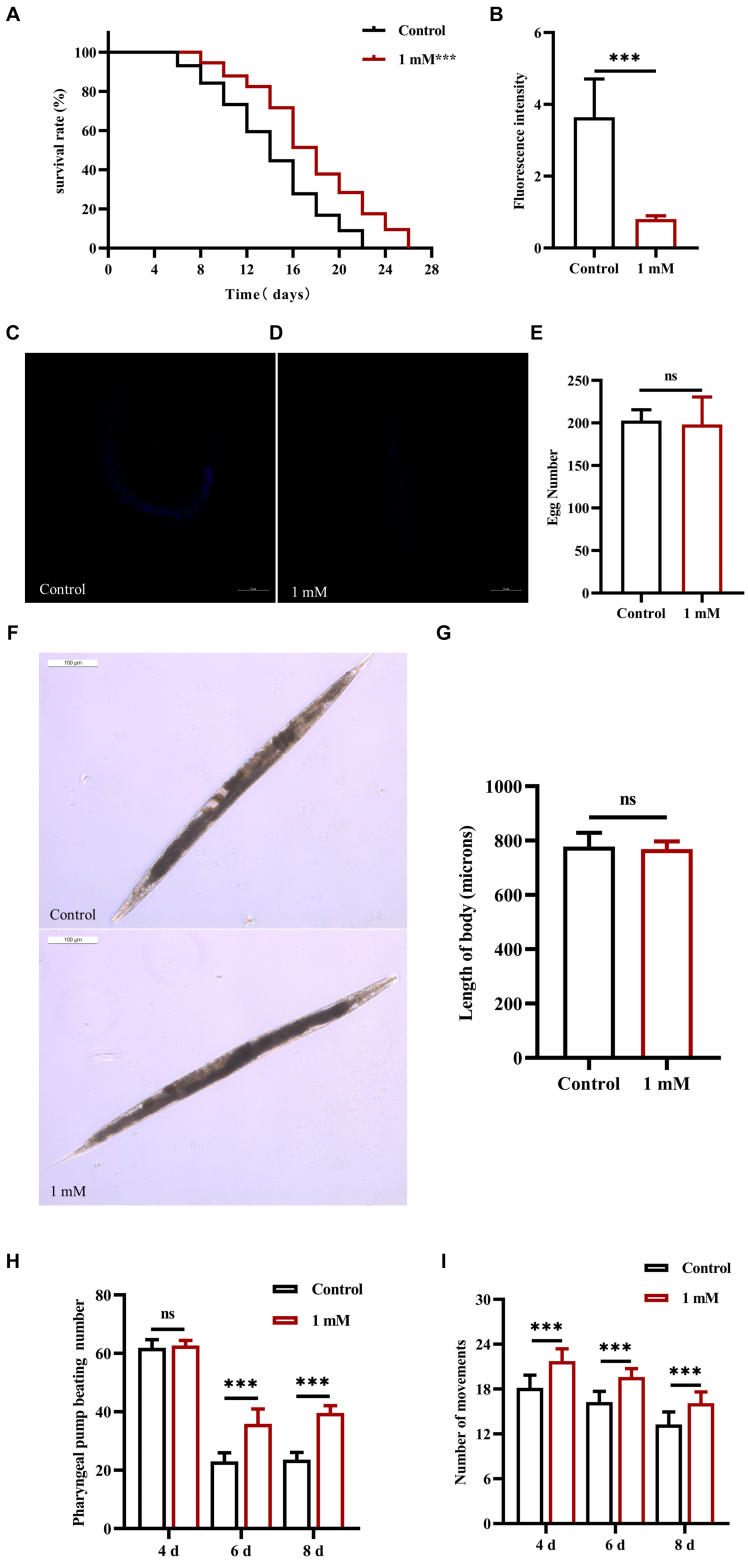
Effect of 4,4′-methylenediphenol on senescence-related indices in worms. **(A)** CL4176 worms were cultured in NGM plates with or without drug (1 mM). The survival curve of the drug-administered group was shifted to the right compared to the Control group, and 4,4′-methylenediphenol prolonged the lifespan of AD worms (*p* < 0.001). **(B)** To investigate whether 4,4′-methylenediphenol inhibits lipofuscin aggregation in worms to delay aging, we observed the autofluorescence of lipofuscin in worms on the 10th day after administration of the treatment. The results showed that the lipofuscin level in worms was reduced after administration of the drug (*p* < 0.001). **(C)** Representative pictures of lipofuscin fluorescence in N2 worms not treated with 4,4′-methylenediphenol. **(D)** Representative pictures of lipofuscin fluorescence of N2 worms in the 1 mM administration group. **(E)** The number of eggs laid by worms in the Control group was 202.800 ± 12.795, whereas the number of eggs laid by worms in the 1 mM administration group was 198.300 ± 32.180, and there was no effect of 4,4′-methylenediphenol administration on the reproductive function of worms (*p* > 0.05). **(F)** Representative pictures of worm body lengths. **(G)** Differences in worm body lengths were not statistically significant (*p* > 0.05). Next, we observed the pharyngeal pump beating frequency and locomotor ability of worms on days 4, 6, and 8 within 30 s. 4,4′-methylenediphenol was able to improve the pharyngeal pump beating and locomotor ability of N2 worms (*p* < 0.001). **(H)** 4,4′-methylenediphenol was able to increase the number of pharyngeal pump beats of worms on days 6 and 8. **(I)** The motility of N2 worms was enhanced on days 4, 6, and 8 after the administration of the treatment.

The lifespan of worms is closely related to senescence, which plays an important role in the development of AD (Santoro et al., 2020). Here, we investigated the effect of 4,4′-methylenediphenol (1 mM) on the lifespan of AD model worms. The maximum lifespan of worms in the Control and 4,4′-methylenediphenol administration group was 22 and 26 days, respectively. Compared with the Control group, the median and mean lifespan of worms in the drug treatment group were significantly prolonged (*p* < 0.05 and *p* < 0.01, respectively), with an average lifespan extension of 20.053%. Survival curves showed a rightward shift after 4,4′-methylenediphenol treatment, indicative of a significantly prolonged lifespan in AD model worms (Table 2; Figure 2A). In addition, lipofuscin accumulates in the intestinal tract of worms as their lifespan increases, so lipofuscin levels are strongly associated with worm aging (Höhn et al., 2010). Lipofuscin fluorescence intensity was stronger in the Control group than in the 4,4′-methylenediphenol administration group, and worms in the former group accumulated significantly higher levels (*p* < 0.001) of lipofuscin in their bodies (Figures 2B–D). The above findings indicated that 4,4′-methylenediphenol can reduce lipofuscin levels in worms, and further suggested that the drug can delay aging in the animals.

**Table 2:** Experimental statistics of CL4176 worm lifespan (x ± s, *n* = 3).

Groups	Maximum lifespan/days	Median lifespan/days	Life expectancy/days	Average life extension rate/%
Control	22	11.940 ± 0.576	14.167 ± 0.629	–
1 mM	26	15.817 ± 0.585**	17.008 ± 0.905*	20.053

The effect of a drug on helminth senescence can be indirectly reflected by the determination of the effect of the drug on the reproductive function of the animals (Hyun et al., 2021). The results showed that the difference in the number of offspring between the Control group and the 1 mM 4,4′-methylenediphenol administration group was not significant (*p* > 0.05), indicating that the drug did not affect the reproductive capacity of the worms (Figure 2E). In addition, measurements of worm body length showed no statistically significant (*p* > 0.05) differences in worm body length between groups on day 2 after drug administration, indicating that the drug had no effect on worm development (Figures 2F,G). Under physiological conditions, the ability of worms to pump their pharynx is indicative of their ability to feed, which, in turn, reflects the extent of senescence (Gao et al., 2015). We found that there was no effect on the twitching ability of the worms on day 4 post-drug administration (*p* > 0.05); in contrast, on days 6 and 8 after treatment, the number of twitching events in the worms of the 4,4′-methylenediphenol administration group was significantly higher (*p* < 0.001) than that of the Control group. The motility of worms also has a large correlation with lifespan (Cogliati et al., 2020). Importantly, locomotor ability is the most basic index reflecting the function of the nervous system and is widely used in the toxicological assessment of drugs and poisons (Wang et al., 2021). Our data showed that, compared with the Control group, the motility of the worms in the treatment group was enhanced on days 4, 6, and 8 post-administration (*p* < 0.001). In conclusion, our findings implied that 4,4′-methylenediphenol enhanced motility in AD model worms, including pharyngeal pump beating and locomotion, and also delayed the aging of the animals (Figures 2H,I).

### 3.3 4,4′-methylenediphenol enhanced stress resistance and reduced ROS levels in N2 worms

**Figure 3:**
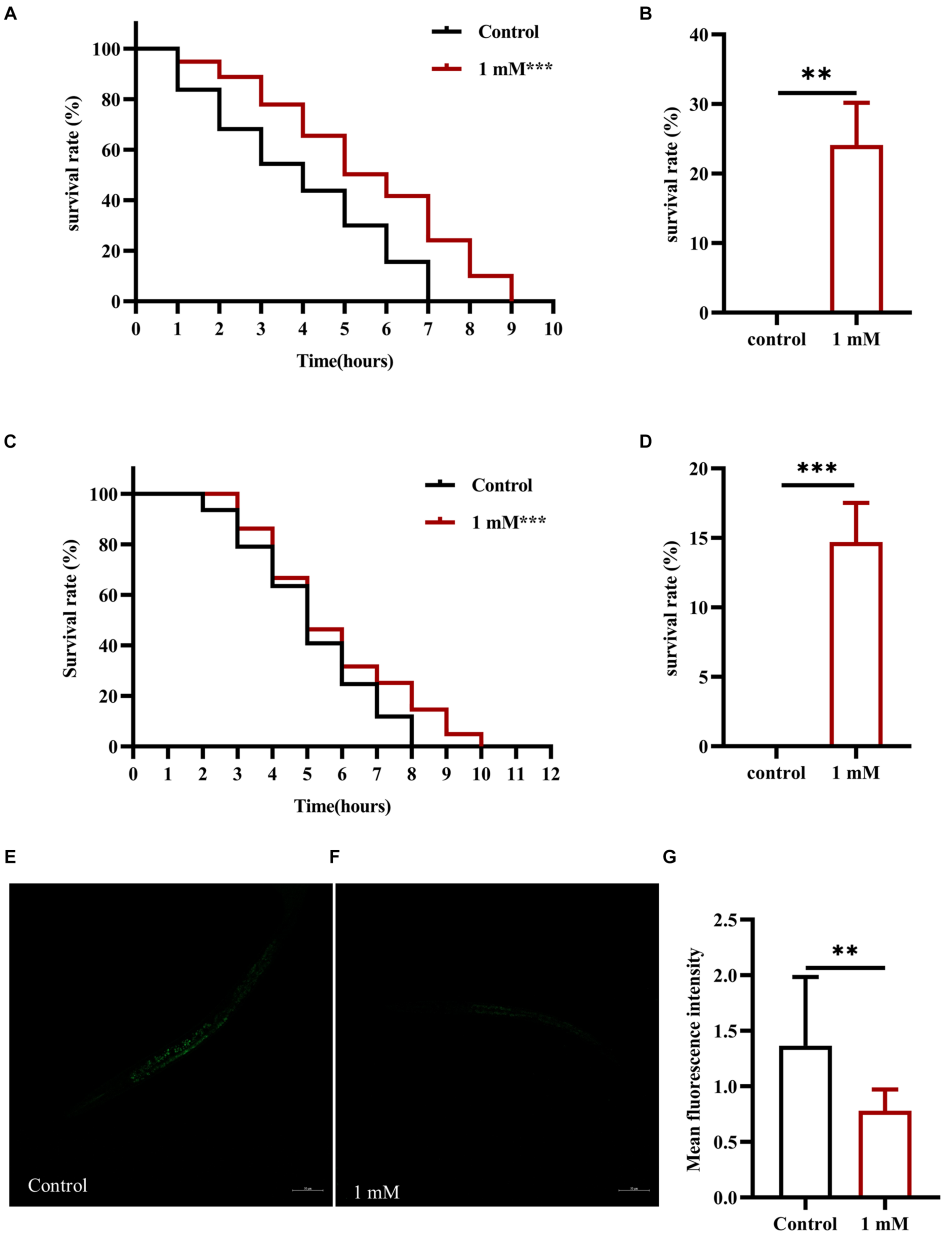
4,4′-methylenediphenol enhances stress resistance and reduces ROS levels in N2 worms. **(A)** Under the high temperature stress at 37°C, the overall lifespan of worms increased after 4,4′-methylenediphenol administration, and the drug enhanced their ability to resist heat stress. **(B)** At the 7th h of heat stress treatment, all worms in the Control group died, and the survival rate of worms in the drug-administered group was 24.138% at this time (*p* < 0.01). **(C)** Juglone (300 μM) was used to construct an oxidative stress damage environment. 4,4′-methylenediphenol administration treatment reduced the oxidative stress damage induced by juglone, and improved the oxidative stress resistance of worms. **(D)** At the 7th h of oxidative stress injury, the survival rate of worms was higher in the drug-administered group compared with the Control group. *p* < 0.01, *p* < 0.001, and the experiment was independently repeated three times. In addition, paraquat (5 mM) was used to induce ROS production in worms, and pictures were taken under a fluorescence microscope after staining with DCFH - DA solution. 4,4′-methylenediphenol reduced ROS levels in N2 worms. **(E)** Fluorescent representative pictures of ROS in N2 worms not treated with 4,4′-methylenediphenol. **(F)** ROS fluorescence pictures of N2 worms treated with 4,4′-methylenediphenol administration. **(G)** ROS fluorescence intensity was measured and analyzed using Image J software.

Stress resistance refers to the ability of an organism to resist a stressful environment and there is a strong positive correlation between increased longevity and enhanced stress resistance (Tan L. et al., 2022). To investigate the effect of 4,4′-methylenediphenol on stress resistance in the nematodes, we first explored the changes in the ability of N2 worms to resist heat stress (37°C) following 4,4′-methylenediphenol administration. The results showed that the overall survival time of worms in the 4,4′-methylenediphenol (1 mM) administration group was prolonged compared with that in the Control group (*p* < 0.001), even after 7 h of administration (*p* < 0.01), indicating that treatment with 1 mM 4,4′-methylenediphenol augmented resistance to heat stress in the animals (Figures 3A,B). Next, to test the ability of the drug to protect against oxidative damage, we exposed N2 worms to juglone (300 μM), a strong oxidant that led to their deaths, and measured the survival rate of the worms. The results showed that compared with the Control group, the survival curve of worms in the 4,4′-methylenediphenol administration group was shifted to the right, and the survival rate was significantly increased (*p* < 0.001). After 7 h of treatment, the survival rates of worms in the Control and drug administration groups were 0 and 14.634%, respectively (*p* < 0.001), showing that 4,4′-methylenediphenol can improve the ability of worms to resist externally induced oxidative stress (Figures 3C,D).

When animals are subjected to a variety of harmful stimuli *in vivo*, ROS production is accelerated, leading to an imbalance between the oxidative and antioxidant systems, which then triggers a series of molecular-mechanical reactions. In AD model animals, it has been shown that these events contribute to Aβ accumulation, which, in turn, enhances ROS production and promotes neuronal damage and death, leading to the occurrence of AD. Accordingly, the determination of ROS levels is crucial in the assessment of the effects of AD-targeting drugs (Song et al., 2022). Here, we used paraquat (5 mM) to induce an increase in ROS contents in AD model worms and found that the average ROS fluorescence intensity in the drug administration group was significantly reduced compared with that in the Control group (*p* < 0.01), indicating that 4,4′-methylenediphenol inhibits the production of free radicals in worms to a certain extent, thereby reducing Aβ-induced toxicity (Figures 3E–G).

### 3.4 4,4′-methylenediphenol reduced Aβ protein deposition in worms

**Figure 4:**
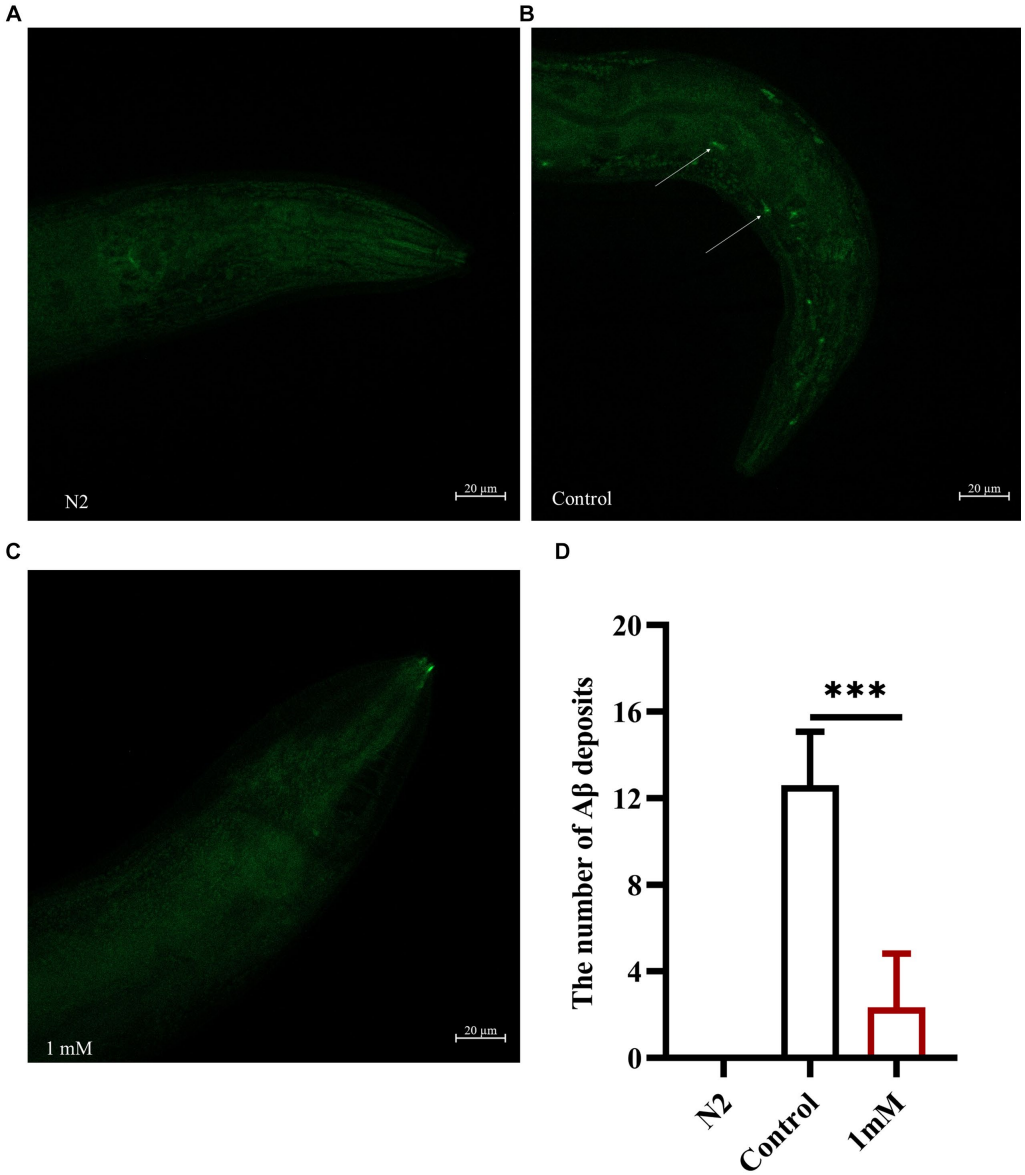
4,4′-methylenediphenol reduces the amount of Aβ protein deposited in transgenic worms CL2006. We measured the amount of Aβ protein deposition by thioflavin S staining. In CL2006 worms, staining with thioflavin S for Aβ protein produces distinct fluorescent patches near the pharynx with white arrows indicating Aβ deposition sites. **(A)** Wild-type N2 worms served as negative controls with no Aβ protein deposition. **(B)** Representative images of CL2006 worms not treated with 4,4′-methylenediphenol, with more deposits. **(C)** Representative images of 4,4′-methylenediphenol-treated worms with significantly less deposits. **(D)** Number of Aβ protein deposits in each group. (*p* < 0.001).

Transgenic *C. elegans* of the CL2006 strain become progressively paralyzed. As the worms age, Aβ gradually accumulates and forms plaques that become toxic, leading to paralysis. Here, we explored the effect of 4,4′-methylenediphenol on the formation of Aβ protein deposits using an *in vivo* Aβ aggregation assay (Fay et al., 1998). Thioflavin S is a homogeneous chemical mixture that can be used for the staining and observation of β-amyloid plaques in AD (DanQing et al., 2021). The results showed that there was no Aβ protein deposition in wild-type N2 worms. In CL2006 worms, however, 4,4′-methylenediphenol (1 mM) administration significantly reduced Aβ protein aggregation in the head of the worms compared with that seen with the Control treatment (*p* < 0.001). These findings revealed that 4,4′-methylenediphenol can reduce Aβ protein deposition in worms, thus minimizing Aβ-induced toxic effects (Figures 4A–D).

### 3.5 4,4′-methylenediphenol affected metabolite abundance in CL4176 AD model worms

**Figure 5:**
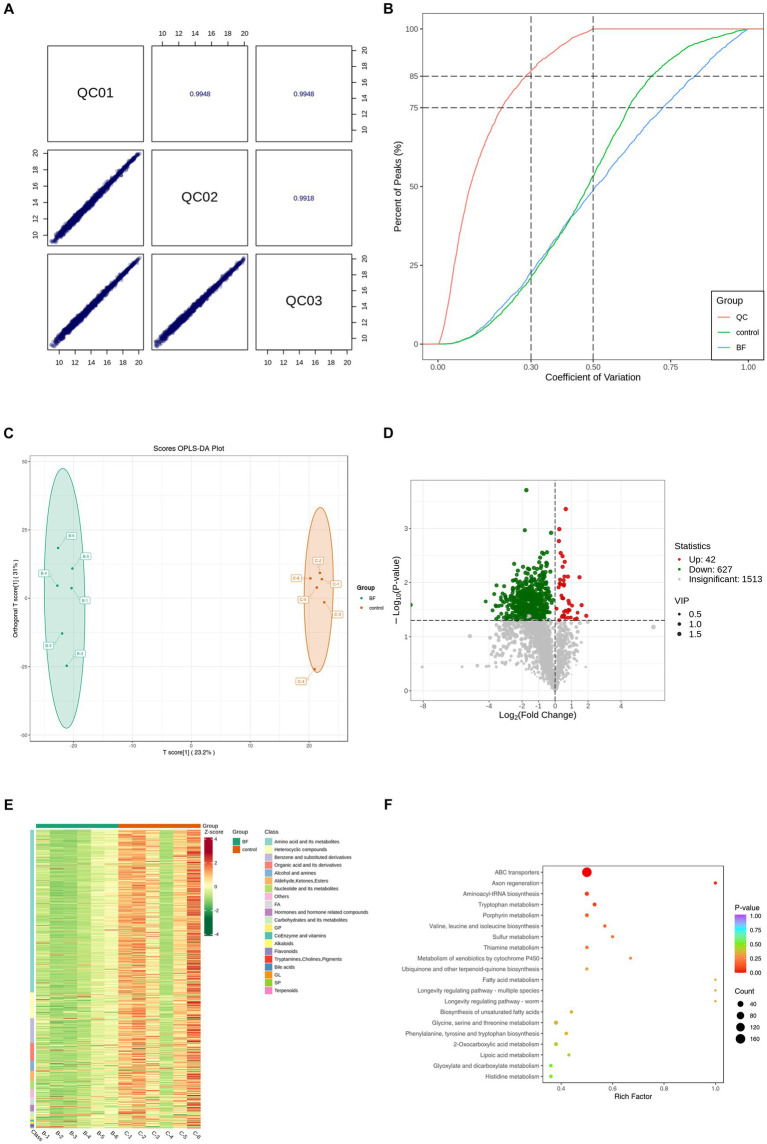
We performed TM wide target metabolome sequencing analysis of AD worms. After synchronization of transgenic AD worms CL4176 to the L1 stage, the worms were cultured on NGM plates with or without drug (1 mM), with no less than 1,000 worms per plate and six parallels per group. After incubation at 16°C for 48 h, the plates were warmed up to 25°C and continued to be incubated for about 30 h. Thereafter, the worms were collected in EP tubes with M9 buffer and washed 2–3 times with sterile water. Sequencing analysis was performed after snap-freezing with liquid nitrogen. **(A)** Pearson correlation analysis was performed on the QC samples, and the higher the correlation of QC samples (| r | is closer to 1) indicates the better stability of the whole detection process and the higher quality of data. **(B)** CV distribution of samples in each group. The proportion of substances with CV value less than 0.5 in QC samples is higher than 85%, indicating that the experimental data are more stable; the proportion of substances with CV value less than 0.3 in QC samples is higher than 75%, indicating that the experimental data are very stable. **(C)** OPLS-DA score plot. Orthogonal Partial Least Squares Discriminant Analysis (OPLS-DA) combines Orthogonal Signal Correction (OSC) and PLS-DA methods, which is able to decompose the X matrix information into two types of information related to Y and irrelevant information, and filter the difference variables by removing the irrelevant differences. **(D)** Differential metabolite volcano plots. Volcano Plot (VP) is mainly used to demonstrate the difference in relative content of metabolites in two groups of samples and the statistical significance of the difference. **(E)** In order to facilitate the observation of the change rule of the relative content of metabolites, we used UV (Unit Variance Scaling) processing on the original relative content of differential metabolites obtained by applying the screening criteria for identification by rows, and plotted the differential metabolite clustering heatmap through the R software package. **(F)** Based on the differential metabolite results, KEGG pathway enrichment analysis was performed to obtain the differential metabolite pathway enrichment map. The closer the *p*-value is to 0, the more significant the enrichment is. The size of the dots in the graph represents the number of differentially significant metabolites enriched into the corresponding pathway. The Rich Factor is the ratio of the number of differential metabolites in the corresponding pathway to the total number of metabolites annotated to that pathway, with larger values indicating greater enrichment. The BF group in all plots is the 4,4′-methylenediphenol (1 mM) treatment group, and all results are for the 1 mM treatment group vs. Control group.

We performed a broad-spectrum TM analysis to investigate the effect of 4,4′-methylenediphenol treatment on the metabolite profile of the AD-like *C. elegans* CL4176 strain. First, Pearson’s correlation analysis was performed on the QC samples, and it was found that the correlation coefficient (*r*) of the QC samples was close to 1, which indicated that the assay process was stable and the data quality was high (Figure 5A). Secondly, the coefficient of variation (CV) distribution plots of all the samples also showed that the percentage of substances with CVs lower than 0.3 in the QC samples was higher than 75%, indicating that the experimental data were stable (Figure 5B). Orthogonal Partial Least Squares Discriminant Analysis (OPLS-DA), which combines orthogonal signal correction and PLS-DA and decomposes X-matrix information into two categories, namely, Y-related and irrelevant, and filters the discrepant variables by removing irrelevant differences (Wang et al., 2022). As shown in Figure 5C, OPLS-DA demonstrated that there was significant separation between the two groups. Next, we screened for differential metabolites using VIP >1 and *p*-value <0.05 as the screening criteria. As shown in the volcano plot in Figures 5D, a total of 669 differentially abundant metabolites were identified, 627 of which were downregulated and 42 upregulated. To facilitate the observation of the relative changes in metabolite content, we generated a heatmap of differential metabolite hierarchical clustering. The first-level classification of the differential metabolites in the clustering heatmap indicated that they were related to amino acids and their metabolites, organic acids and their derivatives, nucleotides and their metabolites, coenzymes and vitamins, alkaloids, flavonoids, and terpenoids (Figure 5E). Finally, we performed KEGG pathway enrichment analysis on the metabolites identified as showing differential abundance (Figure 5F). We found that the top 20 *p*-value-ranked pathways included ABC transporter protein; ubiquinone and other terpene quinone biosynthesis; fatty acid metabolism; unsaturated fatty acid biosynthesis; glycine, serine, and threonine metabolism; and phenylalanine, tyrosine, and tryptophan biosynthesis, among other pathways.

### 3.6 The effects of 4,4′-methylenediphenol on the expression of DAF-16/FOXO, SKN-1/NRF2, GST-4, and SOD-3 in worms

**Figure 6:**
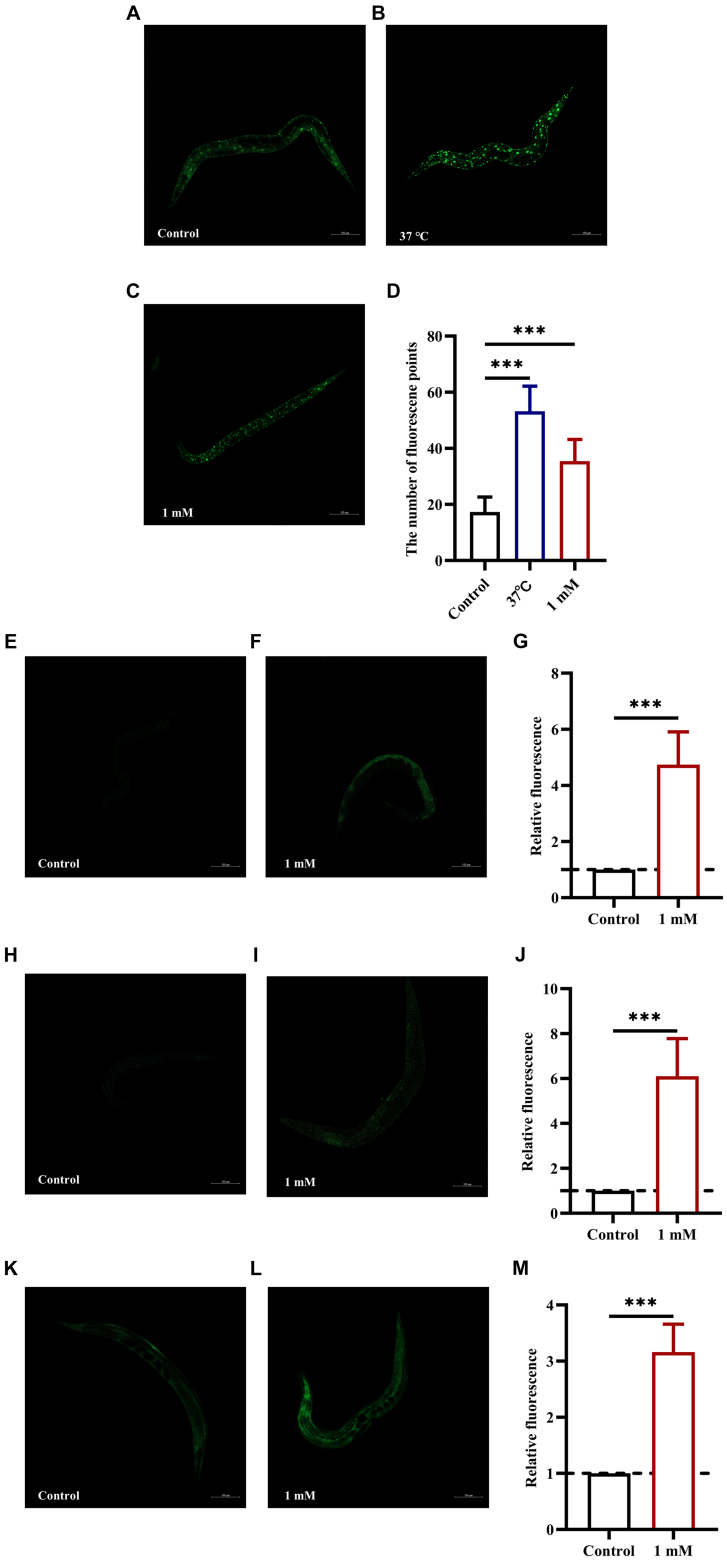
4,4′-Methylenediphenol enhances the expression of DAF-16, SKN-1, SOD-3 and GST-4 in GFP reporter gene worms. Fluorescence images were obtained under a laser confocal microscope, and the green fluorescent spots indicated the nucleus localization of DAF-16 in TJ356 worms treated at 37°C for 30 min as a positive control. Representative images of TJ356 worms: **(A)** Control group. **(B)** Positive control group. **(C)** 1 mM 4,4′-Methylenediphenol group. **(D)** Number of fluorescence points in each group (*p* < 0.001). Representative images of LD1 worms: **(E)** Control group. **(F)** 1 mM 4,4′-Methylenediphenol group. **(G)** Relative fluorescence intensity measurements of SKN-1::GFP in LD1 worms (*p* < 0.001). Representative images of CF1553 worms: **(H)** Control group. **(I)** 1 mM 4,4′-Methylenediphenol group. **(J)** Fluorescence intensity expression measurement of SOD-3::GFP *in vivo* in CF1553 strain in each group. 1 mM 4,4′-Methylenediphenol increased the expression of SOD-3 in worms (*p* < 0.001). Representative images of CL2166 worms: **(K)** Control group. **(L)** 1 mM 4,4′-Methylenediphenol group. **(M)** Relative fluorescence intensity of GST-4::GFP expression in CL2166 worms (*p* < 0.001). Experiments were repeated independently three times.

The insulin signaling pathway plays a key role in the regulation of lifespan. DAF-16 (FOXO transcription factor homolog) is a major regulator of the insulin signaling pathway and its overexpression can delay aging and extend lifespan in worms (Wang et al., 2016). *skn-1*, *gst-4*, and *sod-3* are target genes of DAF-16, and all are associated with longevity and protection against oxidative stress (Liu et al., 2023; Wang et al., 2023). In this study, we first determined the nuclear localization of DAF-16 and the expression of SKN-1. The results showed that treatment with 4,4′-methylenediphenol accelerated the nuclear translocation of DAF-16 in strain TJ356 (*p* < 0.001) (Figures 6A–D), and upregulated SKN-1 expression in LD1 worms (Figures 6E–G). This suggested that the 4,4′-methylenediphenol-mediated mitigation of Aβ toxicity and enhancement of antioxidant properties was, at least partly, due to the activation of DAF-16. Next, we examined the expression of SOD-3 and GST-4. The results showed that 4,4′-methylenediphenol treatment enhanced the fluorescence intensity of SOD-3::GFP in the CF1553 strain (Figures 6H–J), and the fluorescence of the CL2166 strain expressing GST-4::GFP was also significantly increased after 4,4′-methylenediphenol treatment (Figures 6K–M). These results showed that 4,4′-methylenediphenol enhanced the expression of *sod-3* and *gst-4* in the worms (*p* < 0.001), thereby delaying paralysis onset and prolonging the lifespan of the worms.

### 3.7 DAF-16 plays a key role in the neuroprotective effects of 4,4′-methylenediphenol

**Figure 7:**
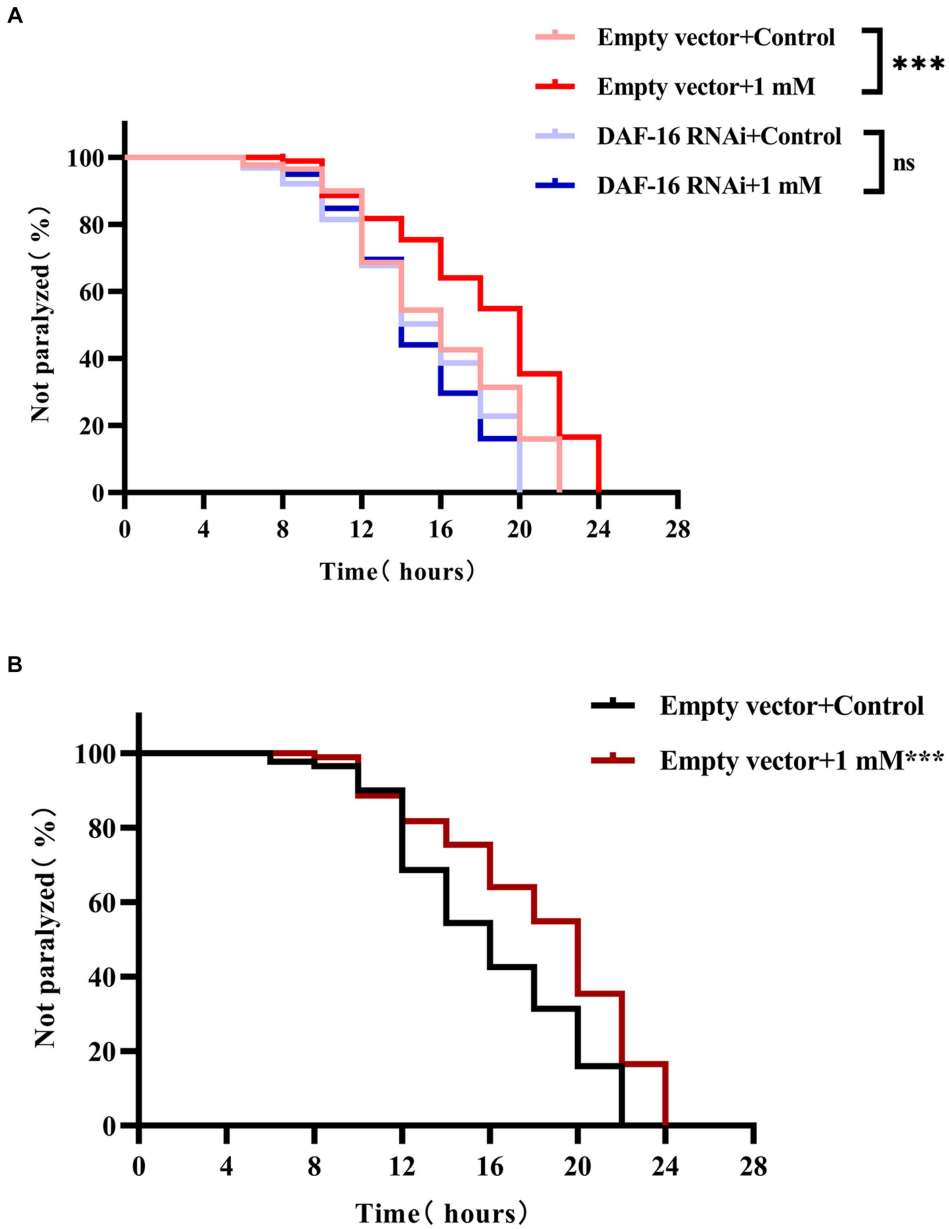
4,4′-methylenediphenol delays worm paralysis by activating DAF-16. **(A)** When the DAF-16 gene was knocked out in the transgenic Hidradenitis elegans cryptic nematode strain CL4176, 4,4′-methylenediphenol lost its ability to alleviate β-amyloid-induced paralysis. DAF-16 expression was knocked down by feeding AD worm CL4176 with *E. coli* strain R13H8.1 bacteria carrying DAF-16 dsRNA. Paralysis curves show that the inhibitory effect of 4,4′-methylenediphenol (1 mM) on worm paralysis disappears after DAF-16 knockdown. **(B)** Whereas control worms without DAF-16 knockdown were significantly delayed from paralysis by 1 mM 4,4′-methylenediphenol treatment. At least 50 worms were tested in each group and the experiment was repeated three times independently.

DAF-16 acts as a major regulator of the insulin/insulin-like growth factor 1 signaling (IIS) pathway and plays an important role in mediating stress resistance, development, reproduction, metabolism, and longevity (Yang et al., 2016; Zullo et al., 2019). Because we found that 4,4′-methylenediphenol activates the nuclear translocation of DAF-16 in TJ356 worms, we next investigated whether the effect of 4,4′-methylenediphenol on paralysis onset in AD model worms was mediated through DAF-16 by knocking down DAF-16 using RNAi. To this end, we performed RNA interference by feeding to knock down DAF-16 in AD worms to verify whether 4,4′-methylenediphenol could continue to delay the paralytic process of the worms. The results showed that when DAF-16 was knocked down in the worms, there was no significant difference in the ability of the 1 mM administration group to delay the paralysis of the worms compared with the Control group (*p* > 0.05). In contrast, the ability to delay Aβ-induced paralysis was significantly enhanced in the 1 mM-administered group in the control group of worms in which DAF-16 was not knocked down (Figures 7A,B). This indicated that DAF-16 plays a crucial role in mediating the neuroprotective effect of 4,4′-methylenediphenol in AD model nematodes.

## 4 Discussion

AD accounts for the highest proportion of dementia cases worldwide and occurs most frequently in older adults. The clinical manifestations of AD include memory loss, cognitive impairment, and impaired self-care ability (Jack et al., 2013). The prevalence and mortality rates of AD and other dementias in China are slightly higher than the global average (Xu et al., 2023), which not only seriously impacts the lives of patients and their families, but also represents a significant economic burden for society (Montgomery et al., 2018; Tahami Monfared et al., 2022). Despite this, currently available therapeutic drugs do not meet the clinical needs of patients or societal demands (Pilonieta et al., 2023).

Natural compounds have shown promise as therapeutic agents for neurodegenerative diseases such as AD (Chen et al., 2021; Li Y. et al., 2024; Ni et al., 2024). Accordingly, several animal models of AD, including mice, dogs, and non-human primates, have been developed aimed at investigating the efficacy and mechanisms of action of natural compounds. However, the high-throughput screening of drugs in these models is complicated due to limitations such as long experimental periods, high costs, and ethical concerns (Jiang and MacNeil, 2023). To overcome these drawbacks, we selected a smaller model organism, *C. elegans*, to investigate the anti-AD therapeutic potential of 4,4′-methylenediphenol. Compared with other model organisms, *C. elegans* has the advantages of small size, low cost, and short experimental period. Furthermore, many AD transgenic worm strains, such as CL2006, CL4176, and CL2166, have been widely used for relevant research applications. These strains can both better mimic Aβ deposition and other pathologic features of AD and more directly simulate the preventive and therapeutic effects of anti-AD drugs (Margie et al., 2013). In addition, *C. elegans* is transparent, allowing neuronal structure and morphology to be tracked through the expression of GFP in specific neurons, and cellular localization can be visualized using GFP-tagged proteins (Chalfie et al., 1994). Moreover, RNAi knockdown strains are available for approximately 86% of *C. elegans* genes, and, combined with the complete genome sequence information, each gene can be targeted in this model. The RNAi-mediated inhibition of target gene expression enables the assessment of the regulatory effect of a drug on both the gene of interest and the associated signaling pathway (Ketting and Plasterk, 2000). The unique advantages of *C. elegans* have allowed its wide use in screening for natural compounds with potential anti-AD effects. For instance, studies based on this model have demonstrated the antioxidant and anti-aging properties of *Lippia origanoides* essential oil, and highlighted its potential for the prevention and treatment of AD (Henrique Moniz et al., 2023).

*Gastrodia elata* is a well-known and valuable medicinal herb with a distinctive odor that is suitable for growing in moist and well-ventilated shady environments, such as Yunnan and Guizhou in China. Studies have shown that *Gastrodia elata* and its extracts have potential for use in the prevention and treatment of AD (Shi et al., 2023a). Indeed, one of the active ingredients of *Gastrodia elata*, p-hydroxybenzyl alcohol, was reported to ameliorate Aβ-induced toxic effects (Liu et al., 2022). However, whether another of its other active ingredients, 4,4′-methylenediphenol, also exerts anti-AD effects has not been investigated. In our preliminary study, we found that 4,4′-methylenediphenol, at an optimal effective concentration of 1 mM, had an ameliorative effect on the paralyzed phenotype of AD model worms, and also prolonged their lifespan. This suggested that this active ingredient may mediate, at least in part, the anti-AD action of *Gastrodia elata*. In a follow-up pharmacodynamic study, we further found that 4,4′-methylenediphenol enhanced the motility of, and was not reproductively toxic toward CL4176 worms. Moreover, it is well known that oxidative stress can exacerbate damage to neuronal cells and thus promote AD pathology (Kam et al., 2013; Mohamed et al., 2021). However, we found that 4,4′-methylenediphenol enhanced the resilience of N2 (wild-type) worms to high-temperature environments, as well as to oxidative stress induced by juglone, and improved resistance to oxidative stress damage, thus increasing their survival rate. In addition, ROS and lipofuscin fluorescence analyses showed that 4,4′-methylenediphenol can reduce ROS contents and lipofuscin levels in N2 worms, resulting in antioxidative stress and anti-aging effects. Aβ is considered a crucial marker of AD pathology in AD-related studies (Paul et al., 2020). Our results showed that 1 mM 4,4′-methylenediphenol treatment led to a significant reduction in Aβ protein deposition in the head of CL2006 worms, which, in turn, reduced Aβ-induced toxicity. Combined, these results suggested that 4,4′-methylenediphenol has considerable antioxidative and anti-Aβ toxicity properties.

The metabolite profile can serve as an indicator of an organism’s phenotype (Favilli et al., 2023) and help to more effectively understand biological processes and their mechanisms. Critically, neurodegenerative disorders, including AD, are mediated by multiple metabolic pathways (Liu et al., 2022). Metabolomics is a high-throughput screening technology that simultaneously detects and quantifies hundreds of thousands of disturbed metabolites (small molecules in the mass range of 50–1,500 Da) in tissues or biofluids, depicting fluctuations in multiple networks affected by a given disease(Huo et al., 2020). Therefore, the qualitative and quantitative analysis of AD worm metabolites after drug administration treatment is essential for elucidating the pathogenesis of AD (Lista et al., 2023). Thus, we subsequently performed a broad-spectrum TM analysis of CL4176 AD model worms to assess the effect of 4,4′-methylenediphenol administration on its metabolites. Using VIP >1 and *p*-value <0.05 as the differential metabolite screening criteria, we identified a total of 669 metabolites that displayed differential abundance between the Control and drug treatment groups, including 42 that were upregulated and 627 that were downregulated. Among them, a pteridine and its derivative, ethionamide, were found to be upregulated after 4,4′-methylenediphenol administration. Treatment with ethionamide, an antibiotic commonly used for the treatment of tuberculosis, enhances the proliferation and migration of mesenchymal stem cells (MSCs), which are a useful source of cells for the treatment of a variety of immune-mediated diseases, including neurodegenerative disorders; however, the poor migratory and survival capacity of MSCs after brain transplantation limit their therapeutic efficacy in the disease microenvironment, and ethionamide pretreatment led to higher survival and enhanced the migratory ability of MSCs in models of a variety of diseases, especially neurodegenerative disorders (Lee et al., 2020). The expression of the metabolite taurodeoxycholic acid (TUDCA) was also significantly upregulated by 4,4′-methylenediphenol. Studies have shown that TUDCA has significant antiapoptotic and neuroprotective activities, effects that are exerted both through the regulation and inhibition of the apoptotic cascade as well as the mitigation of oxidative stress, the protection of mitochondria, and the generation of antineuroinflammatory effects. Moreover, abundant experimental and clinical evidence supports that it may be useful as a disease modifier in the treatment of neurodegenerative diseases (Ochiai et al., 2021; Zangerolamo et al., 2021; Khalaf et al., 2022). Our results showed that 4,4′-methylenediphenol treatment upregulated nicotinamide levels. Nicotinamide, an endogenous PARP-1 inhibitor, has been shown to reduce the levels of oxidative stress, apoptosis, and PARP-1 activity in an Aβ protein-induced rat model of AD, and has therapeutic potential in neurodegenerative processes (Turunc Bayrakdar et al., 2014). TM analysis showed that 4,4′-methylenediphenol also upregulated progesterone contents. Progesterone was reported to significantly inhibit the Aβ-induced activation of the NLRP3 inflammasome in astrocytes by mitigating endoplasmic reticulum stress (Yang et al., 2020). In contrast, the expression of clethodim, an herbicide commonly used in agriculture, was downregulated following 4,4′-methylenediphenol treatment. Studies have shown that clethodim is reproductively toxic to zebrafish and induces neurological dysfunction in this model organism (Wang et al., 2019; Xiong et al., 2019). Methotrexate, which exerts cytotoxic effects through the induction of inflammation and oxidative stress and causes lung damage and hepatotoxicity, was similarly found to be downregulated following treatment with 4,4′-methylenediphenol (Fikry et al., 2023; Rajizadeh et al., 2024). The above observations indicated that the 4,4′-methylenediphenol-induced changes in metabolite abundance have an overall positive effect on neurodegenerative diseases. Our KEGG enrichment analysis also showed that the ABC transporter protein pathway was the most significantly enriched, implying that it may play an important role in mediating the neuroprotective effects of 4,4′-methylenediphenol. ABC transporters comprise a family of integral membrane proteins found in most organisms, and their functions include detoxification and nutrient uptake (Qu et al., 2020). Moreover, relevant studies have shown that ABC transporter proteins are highly expressed in the brain and are involved in the pathology of neurodegenerative diseases; they can also perform tissue-protective physiological functions by reducing or limiting the accumulation of neurotoxins in the brain (Gil-Martins et al., 2020; Katzeff and Kim, 2021). Indeed, the blood–brain barrier serves as one of the major pathways mediating the clearance of Aβ proteins from the brain parenchyma, and ABC transporter proteins play a key role in Aβ cytocytosis at the blood–brain barrier (Zhang et al., 2022). This can attenuate AD pathology by exerting an anti-oxidative stress effect and promoting blood–brain barrier integrity (Abuznait and Kaddoumi, 2012; Menet et al., 2020).

Finally, we used strains TJ356 (DAF-16::GFP), LD1 (SKN-1::GFP), CF1553 (SOD-3::GFP), and CL2166 (GST-4::GFP) to further decipher the mechanism of action of 4,4′-methylenediphenol. The results showed that 4,4′-methylenediphenol not only promoted the nuclear localization of DAF-16, but also enhanced the expression of SKN-1, SOD-3, and GST-4 in worms of the respective strains. Further RNAi analysis revealed that the administration of the drug to worms significantly reduced their paralysis rate, an effect that was not observed when DAF-16 was knocked down. DAF-16, a known nuclear transcription factor that induces transcriptional regulation of many genes involved in aging, development, and stress, is the most widely studied of the factors influencing the lifespan extension in *C. elegans* (Li L. et al., 2024). Meanwhile, SOD-3 is regulated by DAF-16 as a downstream target gene that can reduce ROS levels (Yang et al., 2016). The SKN-1 transcription factor, a direct homolog of mammalian nuclear factor red lineage 2-related factor 2 (Nrf-2), has also been associated with longevity and oxidative stress responses (Tullet et al., 2017). Gst-4, a downstream target gene of DAF-16 and SKN-1/Nrf2, improves the body’s ability to resist oxidative stress-induced injury (Kahn et al., 2008). In summary, 4,4′-methylenediphenol exerts neuroprotective effects by activating the DAF-16/FOXO and SKN-1/NRF2 pathways, thereby reducing Aβ-induced toxicity in AD worms.

In conclusion, our results indicated that 4,4′-methylenediphenol enhances stress resistance in *C. elegans* by activating multiple cytoprotective pathways, possesses antioxidant and anti-aging activities, and has ameliorative effects on Aβ protein-induced toxicity, which, in turn, delays the onset of AD-like symptoms. In addition, 4,4′-methylenediphenol positively influences the metabolite profile of AD model worms and is expected to be a potential anti-AD drug candidate. However, the pathogenesis of AD is complex and regulated by multiple factors, and the anti-AD effects of 4,4′-methylenediphenol have not been investigated at the level of genes and transcription factors. In future studies, we will explore the targets and mechanisms of action of this active ingredient using a combination of transcriptomics and qPCR. We also aim to screen for genes and signaling pathways related to Aβ toxicity and oxidative stress in worms to further elucidate in-depth the mechanisms of anti-AD action of 4,4′-methylenediphenol.

## Data availability statement

The original contributions presented in the study are included in the article/Supplementary material, further inquiries can be directed to the corresponding author.

## Ethics statement

The manuscript presents research on animals that do not require ethical approval for their study.

## Author contributions

XY: Conceptualization, Data curation, Formal analysis, Project administration, Software, Writing – original draft, Writing – review & editing, Investigation, Methodology, Validation, Visualization. JT: Conceptualization, Formal analysis, Investigation, Methodology, Writing – original draft. TX: Conceptualization, Formal analysis, Methodology, Writing – original draft, Validation. XD: Conceptualization, Formal analysis, Writing – original draft, Data curation, Funding acquisition, Project administration, Resources, Software, Supervision, Writing – review & editing.

## Footnotes

^1^ExionLC AD, https://sciex.com.cn/

^2^QTRAP, https://sciex.com/
